# Utilization of reproductive health services among undergraduate regular class students in Assosa University, BGRS: a comparison among three varieties of multilevel binary logistic regression models

**DOI:** 10.1186/s12913-024-11123-8

**Published:** 2024-05-26

**Authors:** Molalign Gualu Gobena, Yihenew Mitiku Alemu

**Affiliations:** 1 Assosa University, Assosa, Ethiopia; 2 Injibara University, Injibara, Ethiopia

**Keywords:** Reproductive health services, Random effect, Multilevel binary logistic regression model, Random intercepts, Random slopes, Hierarchical structure, Fixed effect

## Abstract

**Introduction:**

Reproductive health service (RHS) helps for people to have a delighted and safe sex through their life journey. It enables especially for women to go safely through pregnancy and childbirth and provide couples with the best chance of having a healthy infant. Therefore, this study aimed to identify the significant determinants of RHS utilization among undergraduate regular class students in Assosa University by using advanced methodology.

**Methods:**

We used cross-sectional study design to collect RHS data from 362 students in Assosa University from 5 to 16, may 2021. These students were selected using stratified random sampling technique. We also used cross-tabulation to summarize the extents of RHS utilization across all predictors in terms of percentage and three varieties of multilevel binary logistic regression model to model the determinants of RHS.

**Results:**

42.27% of undergraduate regular class students in Assosa University utilize at least one type of RHS during their time at Assosa University whereas, 57.73% of undergraduate regular class students in this University are not utilized it. Among three varieties of multilevel binary logistic regression models, the random slopes two-level model was selected as a best fitted model for the datasets. At 5% level of significance, awareness about RHS, gender, preference of service fees and student’s monthly average income were significant predictor variables in this model. In addition, the covariates; age, gender and preference of service fees have a significant random effects on utilization of RHS across all colleges/school.

**Conclusion:**

Students who; preferred service fee as usual rate, have awareness about RHS, are females and have high monthly average income were more likely to utilize RHS. RHS utilization among undergraduate regular students in Assosa University is likely to increase more effectively with interventions that address these factors.

**Supplementary Information:**

The online version contains supplementary material available at 10.1186/s12913-024-11123-8.

## Introduction

Reproductive health service (RHS) helps for people to have a delighted and safe sex through their life journey. Every men and women have the right to be informed about reproductive health and the right of access to appropriate reproductive health services. This will enable especially for women to go safely through pregnancy and childbirth and provide couples with the best chance of having a healthy infant [1]. Reproductive health service is defined as the constellation of methods, techniques and services that contribute to reproductive health and wellbeing by preventing and solving reproductive health problems [2, 3]. It includes education service, improving antenatal, delivery, postpartum and newborn care; providing high-quality services for family planning, including infertility services; eliminating unsafe abortion; combating sexually transmitted infections, including HIV, reproductive tract infections, cervical cancer and other gynecological morbidities; and promoting sexual health [1, 4, 5].

The disproportionate number of young people who experience life-threatening reproductive health complications each year is a worrying global trend. As compared to other age groups, this issue is especially concerning, according to a study by [6]. These issues highlight the need for better access to reproductive healthcare services and education because they seriously jeopardize young people’s wellbeing.

In 2016, Ethiopia’s RHS utilization rate in four types of RHS were 37.5% in health facility deliveries, 37.2% in skilled delivery assistance, 36.3% in 4 + antenatal visits and 20.4% in use of modern contraception, which are highly lower than the East and Central African rate [7]. It’s crucial to remember, though, that Ethiopia has seen a steady rise in the use of RHS in recent years. For instance, in 2019 the utilization rate in four types of RHS were 48% in health facility deliveries, 50% in skilled delivery assistance, 43% in 4 + antenatal visits and 41% in use of modern contraception, thus the rise in just four years is noteworthy [7–9]. In Ethiopia, over the period of 12 years (2005–2016), the rates of RHS utilization in four RHS were significant. Moreover, over this period the percentage of change in health facility deliveries, skilled delivery assistance, 4 + antenatal visits and use of modern contraception are 25.2%, 24.5%, 19% and 9.5% respectively [7]. RHS have become more widely used in Ethiopia as a result of a number of factors, including improved access to care, especially in rural areas, decreased costs, and increased government investment. Raising awareness of the availability and significance of reproductive health services has also contributed to this advancement. Even with the advancements, there are still large regional differences in the availability and use of reproductive health services in Ethiopia [10–12]. More studies at various settings should be done to guarantee that every young people have access to the high-quality RHS they require, regardless of their geography, wealth, or social standing [3, 13–15]. But, still some studies regarding the utilization of RHS and its determinants were conducted in different part of Ethiopia [15–18].

The official school age interval of tertiary education in Ethiopia is 19–23 [19]. This indicates that young and youth people make up the great majority of university students. University students in Ethiopia may be more likely to become unintentionally pregnant, contract sexually transmitted diseases (STDs) like HIV/AIDS, have unsafe abortions, and experience limited sexual and reproductive health education and resources due to factors specific to this age group [20].

Ethiopia has seen a notable surge in university enrollment, with a rise in students from 447,693 in 2010/11 to 593,571 in 2013/14 [19]. Due to this quick growth, a sizable fraction of students are in the “youth” and “young” age range. This age group is recognized to be more vulnerable to risks related to sexual and reproductive health (SRH) because of things like insufficient experience and education. Although data indicates an increase in the number of students [19], research indicates that universities have not kept up with the demand for access to and knowledge about critical Reproductive Health Services (RHS) [21]. Empirical techniques have frequently been used in prior research on RHS utilization to look into potential influencing factors [19]. Nevertheless, by using a more sophisticated methodology, this study seeks to close this gap. Additionally, the adjustment to university life brings with it special opportunities as well as difficulties that may affect students’ requirements for sexual and reproductive health [21].

However, knowledge of the factors that influence reproductive health decisions can help university health provider, policymakers, and educators create interventions that are tailored to the needs of undergraduate regular class students. Hence, this study aimed to identify the significant determinant of RHS utilization among undergraduate regular class students in Assosa University.

## Methods

### Study setting and design

Assosa University is situated in BGRS capital city, Assosa town. It is situated 660 km from Addis Ababa, 96 km east of the Ethiopian-Sudanese border, and 210 km south of the Grand Ethiopian Renaissance Dam. Its founding goals were to produce highly skilled and committed workforce, carry out research, and perform community service. Currently, the university is running both undergraduate and graduate program in seven colleges and one school.

In this study, we have used cross-sectional study design to collect RHS data from students in Assosa University from 5 to 16, may 2021. The data were collected using self-administered questionnaire (it is attached as a supplementary file). The questionnaire was developed by our team. We followed a rigorous process to ensure its reliability and validity. This included reviewing relevant academic research on RHS utilization among university students in Ethiopia [5, 14, 22, 23], consulting with experts in the field of sexual and reproductive health, and pre-testing the questionnaire with a small group of students at Assosa University.

To minimize errors and bias in data recording and manipulation, we implemented key procedures like pilot testing the questionnaire (reducing measurement error), training data collectors (minimizing interviewer bias), double data entry (mitigating transcription errors), and range checks (identifying potential recording errors).

### Variables

Utilization of RHS is the outcome variable in this study. It has two possible responses which are coded as 1 if students utilize it otherwise coded as 0. Independent variables in this study are age (its coded value; 0 = 18–20, 1 = 21–23, 2 = > 23), awareness about RHS (its coded value; 1 = yes, 0 = no), parents’ occupation (its coded value; 0 = formal employee, 1 = farmer, 2 = casual laborer, 3 = self-employee), gender (its coded value; 0 = male, 1 = female), students’ monthly average income (its coded value; 0 = < 250 ETB, 1 = 251–500 ETB, 2 = 501-1,000 ETB, 3 = 1,001–1,500 ETB, 4 = > 1,500 ETB), place of residence (its coded value; 0 = urban, 1 = rural), preference of service fees for RHS (its coded value; 0 = At usual rate, 1 = with discount, 2 = free of charge), parents’ monthly average income (its coded value; 0 = < 2,500 ETB, 1 = 2,501-4,000 ETB, 2 = 4,001–5,000 ETB, 3 = 5,001–10,000 ETB, 4 = > 10,001 ETB) and religion (its coded value; 0 = Orthodox, 1 = Muslim, 2 = Protestant, 3 = Catholic, 4 = Other). We have considered students to be RHS users if they utilize at least one type of RHS during their time at Assosa University; if not, we have considered them to be non-users.

### Sampling design

We can assume that the students in Assosa University are varying in utilizing reproductive health service from college/school to college/school which implies heterogeneity. Therefore, one of appropriate sampling technique for heterogeneous population is stratified sampling [24]. Hence, in this study we use stratified random sampling. With stratified sampling, the population is divided into homogeneous, mutually exclusive groups called strata, and then independent samples are selected from each stratum [25]. Any of the sample design can be used to sample with in strata, from the simpler method such as Simple Random Sampling (SRS) or Systematic Sampling (SYS) to the more complex methods such as Population Proportion to Size (PPS), cluster, multi-stage or multi-phase sampling [24]. The sample in this study is stratified. Students were stratified by college/school, which yielded 8 sampling strata. Samples of students were selected independently in each stratum (college/school). A total of 362 students were selected with probability proportional to the college/school size and with independent selection in each sampling stratum (college/school) with the sample allocation given in Table 1. The college/school size is the number of students in each college/school as determined in the record of Assosa University registrar office. The students with in each college/school serve as the sampling unit for the selection of students. The data collectors distribute the questionnaire only for those pre-selected students. No replacement or change of the pre-selected students will not allow in the implementing stages to prevent bias. All students who are usual members of the selected college/school were eligible for this study. By pivot survey, $p=0.5,q =0.5, \alpha =5\% ,d=5\%$, we can determine the required number of students in each college/school using the following formula.

$${n}_{0}=\frac{{z}_{\alpha /2}^{2}pq}{{d}^{2}}$$

If $\frac{{n}_{0}}{N}<5\%$, then we will use $n={n}_{0}$ otherwise we will use $n=\frac{{n}_{0}}{1+\frac{{n}_{0}}{N}}$,

where, ${n}_{0}$ =is initial sample size (number of students) in each college/school, P is proportion of student who is utilizing reproductive health service, q is proportion of students who is not utilizing reproductive health service, d=margin of error, $\alpha$ =level of significance, n is the selected number of students in each college/school, N is the number of students in Assosa University. Accordingly, by proportional allocation we will get sample size as it illustrated in the following table. ${n}_{h}=\frac{{N}_{h}}{N}$ where, ${n}_{h}$=the selected number of students in each college/school, ${N}_{h}$ is the number of students in each college/school.

Here, $${n}_{0}=\frac{{1.96}^{2} \times 0.5\times 0.5}{{0.05}^{2}}=384.16$$

hence $$\frac{{n}_{0}}{N}=\frac{384.16}{6298}=0.061>5\%$$,

which implies $$n=\frac{{n}_{0}}{1+\frac{{n}_{0}}{N}}=n=\frac{384.16}{1+0.061}=362$$

**Table 1:** The selected number of students and number of students in each colleges/school

h	Colleges/school	${N}_{h}$	${n}_{h}$	h	Colleges/school	${N}_{h}$	${n}_{h}$
1	Natural and computational Sciences	644	37	5	Agriculture	584	33
2	College of Engineering	2453	141	6	Computing & Informatics	450	26
3	Business & Economics	892	51	7	Social sciences & humanity	573	33
4	Health sciences	482	28	8	School of Law	220	13

Here also, N=${N}_{1}$+${N}_{2}$+${N}_{3}$+${N}_{4}$+${N}_{5}$+${N}_{6}$+${N}_{7}$ +${N}_{8}$ =644+2453+892+482+584+450+573+220=6298 and n=${n}_{1}$ +${n}_{2}$ +${n}_{3}$ +${n}_{4}$ +${n}_{5}$ +${n}_{6}$ +${n}_{7}$ +${n}_{8}$ = 37+141+51+28+33+26+33+13=362.

### Methods of data analysis

We used both descriptive and inferential methods of data analysis to analysis the collected data. First, we used cross-tabulation to provide the descriptive statistics. In this analysis, the extents of RHS utilization across all predictors in terms of percentage were summarized. Second, we used a multilevel version of binary logistic regression model (i.e., multilevel binary logistic regression model) to model the determinants of RHS. This model helps to identify both fixed effect and random effects. Consequently, the true effect of predictors across all colleges/school and response variation at different college/school were clearly indicated.

### Multilevel modeling for stratified data

Data sets in this study have a hierarchical structure. For example, students are nested within their respective college/school. This is clearly reflected in our used sampling design which is called stratified random sampling. Even if this sampling technique provides optimum sample size, in multilevel structure case it leads to dependency among observations within strata (e.g., college/school). One of the preliminary assumptions of using conventional regression model is the validation of independent assumption among observations. In other words, this model no longer applied for data with hierarchical nature. Failing to account for the above assumptions during the analysis phase and using the empirical regression model have an impact on the precision of parameter estimates and their standard errors. This is also having a direct implication on their significant effects. To avoid the confusion in latter description of this hierarchical data structure, we assumed students as “lower level (first), college/school as “higher level (second)” and strata as “group/cluster”. In the phenomena of hierarchical data, variables may be defined at different hierarchical level and this cannot be hosted under empirical regression model. These all problems addressed by the virtue of multilevel modeling. Modeling the effect of individual level factors, group level factor and their collective impacts on the dependent variable of interest is called multilevel modeling.

In our used dataset, grouping students based on their colleges/school may have a contextual impact on the outcome of interest (e.g., Utilization of RHS) which leads to have a correlated response of students within college/school. Moreover, even when the measurements of the characteristics of the selected students are the same, the outcomes of two randomly selected students from the same college/school may be more similar than the outcomes of two randomly selected students from different college/school. These issue handled by the use of Multilevel modeling [26–28]. Hence, multilevel binary logistic regression model can be used to simulate the relationship between a dichotomous response variable and a group of independent variables while taking the data’s nested structure into account [29].

### Model building and selection process

The overall aim in this section is to progressively build a more complex model that captures the hierarchical structure of the data and identifies significant factors influencing RHS utilization among students at Assosa University. Hence, we have undergone three processes to come up with the final best fitted model for the dataset in this study. First, we made population proportion test among college/school to know whether there is a significance difference between college/school or not. This is helpful because it helps to identify whether the data needs multilevel data analysis or single level data analysis. The test result, however, assured to use multilevel data analysis (refer Population Proportion Heterogeneity (**PPH) Test** from result section). Second, we ran random effect univariate model for each individual covariates to know whether they have a significant (p-value < 0.10) random effects across college/school or not. Moreover, it is a precondition to fit a random slopes two-level model for the dataset. Third, we analyzed the data by considering three variety of multilevel binary logistic regression model (e.g., null two-level model, random intercepts two-level model and random slopes two-level model) and then we used their AIC score to select the best fitted model for the dataset. The detail of the above all multilevel models described in [26, 27, 29].

## Results

In this section, we have presented both descriptive and inferential statistics outputs. Entirely, the analysis was done using R-4.3.2 statistical software.

### Descriptive statistics

The result shows that 42.27% of undergraduate regular students utilize at least one type of RHS during their time at Assosa University whereas, 57.73% of undergraduate regular students are not utilized it.

**Table 2:** Cross tabulation of RHS with predictor variables

Variable	Category	Utilization of RHS		Total (%)	Chi-sqr	df	P-Value
		No	Yes				
		Count (%)	Count (%)				
Awareness	No	93 (25.7%)	12 (3.3%)	105 (29%)	72.76	1	< 0.001
	Yes	116 (32%)	141 (39%)	257 (71%)			
Parents’ monthly average income	< 1,000 ETB	37 (10.2%)	21 (5.8%)	58 (16%)	11.15	4	0.025
	1,000–2,500	26 (7.2%)	39 (10.8%)	65 (18%)			
	2,501-5,000	36 (9.9%)	23 (6.4%)	59 (16.3%)			
	5,001–7,500	25 (6.9%)	12 (3.3%)	37 (10.2%)			
	> 7,500 ETB	85 (23.5%)	58 (16%)	143 (39.5%)			
Preference of service fees for RHS	At usual rate	39 (10.8%)	60 (16.6%)	99 (27.4%)	22.71	2	< 0.001
	With discount	73 (20.2%)	28 (7.7%)	101 (27.9%)			
	Free of charge	97 (26.8%)	65(18%)	162 (44.8%)			
Students’ monthly average income	< 250 ETB	44 (12.2%)	61 (16.9%)	105 (29.1%)	17.34	4	0.002
	251–500 ETB	68(18.8%)	40 (11%)	108 (29.8%)			
	501–1000 ETB	58 (16%)	25 (6.9%)	83 (22.9%)			
	1001-1500ETB	14 (3.9%)	8 (2.2%)	22 (6.1%)			
	> 1500 ETB	25 (6.9%)	19(5.2%)	44 (12.1%)			
Parent’s occupation	Formal employees	45 (12.4%)	34 (9.4%)	79 (21.8%)	15.59	3	0.001
	Farmer	92 (25.4%)	90 (24.9%)	182 (50.3%)			
	Casual laborer	29(8%)	5 (1.4%)	34 (9.4%)			
	Self-employees	43(11.9%)	24 (6.6%)	67 (18.5%)			
Place of residence	Urban	104 (28.7%)	73(20.2%)	177 (48.9%)	0.15	1	0.70
	Rural	105 (29%)	80 (22.1%)	185 (51.1%)			
Religion	Orthodox	129 (35.6%)	97 (26.8%)	226 (62.4%)	10.61	4	0.031
	Muslim	25 (6.9%)	26 (7.2%)	51 (14.1%)			
	Protestant	44 (12.2%)	19 (5.2%)	63 (17.4%)			
	Catholic	8 (2.2%)	3 (0.8%)	11 (3%)			
	Others	3 (0.8%)	8 (2.2%)	11 (3%)			
Gender	Male	167 (46.1%)	74 (20.4%)	241 (66.5%)	39.48	1	< 0.001
	Female	42 (11.6%)	79 (21.8%)	121 (33.4%)			

Undergraduate regular class students utilize at least one type of RHS during their time at Assosa University at the minimum age of 18 years and at the maximum age of 35 years. The percentage of undergraduate regular class students of Assosa University who were utilized at least one type of RHS during their time at Assosa University were higher for those who having awareness about RHS (39%) than those who have not (3.3%). Accordingly, the interpretation for other predictors can be provided (Table 2).

We were used chi-square test of association to see the linear association between each independent variables and dependent variable. With this connection, the p-value for each linear association test from the above Table 2 indicate that except the variable place of residence (P-value = 0.700 > 0.05) all variables that we have considered in this study have a linear association with utilization of RHS.

### Population Proportion Heterogeneity Test (PPH Test)

We used a chi-squared test to verify that whether this hierarchical data nature calls for a multilevel data analysis or not. The chi-squared test for proportion of utilizing RHS heterogeneity across colleges/school provides X-squared = 32.502, df = 7, p-value = 3.277e-05 which is significant at 5% level of significant. Therefore, this implies that the proportion of utilizing RHS among undergraduate regular class students is differed on at least between two colleges/schools. This is also an implication to use a multilevel data analysis for this dataset.

### Test for random effects of covariates across groups

We used likelihood ratio test to verify whether each covariate have a significant random effects on utilization of RHS across groups (college/school) or not at 10% level of significance. Then after, we included those significant variables into the random component of a two-level random slope model to fit the dataset (Table 3). Model 3 were built with the consideration of this notion (Table 4). The test used to compare two nested models, the random intercept (reduced model) versus the random slope model (i.e., full model). The full model possesses the random effect of covariates, while reduced model doesn’t possess the random effect of covariates. This test is an implicit test for random effects of covariates across groups. For example, if the test result rejects the null hypothesis, it means that the random slope model is a better fit of the data. In other ways, it suggests that the covariate varies across colleges/schools.

**Table 3:** Test for random effects of covariates on Utilization of RHS across colleges/schools

Tested variable	AIC score for;				
	Reduced Model	Full Model	Chisq	Df	*P*-value
Age	485.81	485.19	4.6105	2	**0.0997**
Awareness of RHS	417.69	419.76	1.9277	2	0.3814
Parents’ occupation	475.35	489.04	4.3006	9	0.8905
Gender	455.35	421.60	37.7480	2	**< 0.0001**
Parents’ monthly average Income	479.68	499.82	7.8629	14	0.8963
Place of residence	485.83	489.80	0.0263	2	0.9869
Preference of service fees for RHS	467.88	467.18	10.701	5	**0.0576**
Students’ monthly average	485.04	509.21	3.8328	14	0.9964
Religion	483.30	504.17	7.1332	14	0.9295

The test results shows that the covariates; age, gender and preference of service fees for RHS have a significant random effects across colleges/schools (P-value < 0.10). Specifically, it means that the considered model permits the difference in age, gender and preference of service fees for RHS among undergraduate class students within colleges/schools to differ across colleges/schools (Table 1). Therefore, we have considered these variables to fit a two-level random slope models from which our conclusion is based (Table 3).

### Results from three varieties of multilevel binary logistic regression model

In this analysis, we have fitted three variety of multilevel binary logistic regression model namely; null two-level model (model 1), random intercepts two-level model (model 2) and random slopes two-level model (model 3). At 5% level of significance, the intercepts in model 2 and 3 are significant while it is insignificant in model 1. The insignificancy of intercepts in model 1 implies that the overall probability of utilizing RHS among undergraduate regular class students in Assosa University is not significantly differ from 50%. This means that we have no supportive evidence for the uniformity prevalence of RHS utilization among undergraduate regular class students across all colleges/schools.

The intercept in model 2 is negative (-5.6381). This reveals that the overall probability of utilizing RHS among undergraduate regular class students in Assosa University is less than 50%.

This indicates that only small proportions of students in all colleges/schools are expected to utilize RHS on average. In other way, the significant intercept in this model clearly indicates that grouping students based on colleges/schools related with a significance difference in the overall likelihood of utilizing RHS across all colleges/schools. But, significant intercept in model 3 can be treated in two ways. The first way is similarly interpreted as model 2 i.e. across all colleges/schools, on average, the overall likelihood of RHS utilization among undergraduate regular class students in Assosa University is statistically significance. The second way imply that the effect of each covariate in the likelihood of utilizing RHS across colleges/schools is different. Moreover, grouping students based on colleges/schools is linked to a significant variation in the relationship between the covariates and utilization of RHS across colleges/schools as well as the overall probability of utilizing RHS. The negative intercept in this model have similar interpretation as negative intercept in model 2.

In general, the insignificancy of intercept in model 1 but significant of it in model 2 and 3 can be viewed as the variation in utilization of RHS across all colleges/schools is masked when the grouping factor (colleges/schools) is the only component in the model. Hence, the inclusion of the random intercepts and consideration of random slope makes it possible to identify group-level variations that the null two-level model was unable to show (Table 4).

**Table 4:** Three variety of multilevel binary logistic regression models for Utilization of RHS in 8 colleges/schools

No	Variable Categories	Model 1		Model 2		Model 3	
		Coefficient (95% CI)	p-value	Coefficient (95% CI)	p-value	Coefficient (95% CI)	p-value
**Fixed Effects**							
	intercept	-0.2566 (-1.2450,0.7318)	0.318	-5.6381(-8.4196, -2.8566)	**0.0056**	-5.7015 (-8.9446, 2.4583)	**0.0393**
1	Age			0.1141(-0.4216,0.64968)	0.1306	0.0779 (-0.5736, 0.7295	0.4851
2	Awareness(Yes)			2.8227(1.5686, 4.0769)	**<0.0001**	3.2565 (1.8639,(4.6491)	**<0.0001**
3	farmer			0.4428(-0.7934, 1.6791)	0.2706	0.7349 (-0.6393,2.1090)	0.1389
	Causal laborer			-1.5658(-3.2310, 0.0995)	**0.0318**	-1.3769 (-3.3703,0.6165	0.1877
	Self-employee			-0.0297(-1.3428, 1.2835)	0.9478	0.2580 ( -1.1741,1.6901)	0.6324
4	Gender(Female)			1.6254(0.5028,2.7481)	**<0.0001**	2.8215 (0.8747,4.7684)	**0.005**
5	2,501-4,000ETB			-0.5802(-1.7960, 0.6355)	0.1355	-0.5006 (-1.7754,0.7742)	0.2415
	4,001-5,000ETB			-0.3003(-0.9770,1.5776)	0.4840	0.0645 (-1.3278,1.4569)	0.8993
	5,001-10,000 ETB			-0.9242(-2.4908, 0.6423	0.1520.	-0.9473 (-2.6615,0.7668)	0.2202
	>10,001 ETB			-0.1250(-1.5251, 1.2750	0.8084	0.1581 (-1.3246,1.6408)	0.7845
6	Residence (Rural)			0.1321(-0.9837, 1.2478)	0.6867	0.0686 (-1.1294,1.2665)	0.8558
7	With discount			-1.2702(-2.5091, -0.0313)	**0.0017**	-1.8739 (-3.9542,0.2064)	0.0997
	Free of charge			-0.7653(-1.8970, 0.3665)	**0.0231**	-1.5117 (-3.1926,0.1691)	**0.0419**
8	251-500 ETB			1.6280(0.2161, 3.03981)	**0.0019**	2.2592 (0.7107,3.8077)	**0.0003**
	501-1000 ETB			0.9006(-0.5142, 2.3155)	0.0871	1.0404 (-0.5050,2.5857)	0.0976
	1001-1500 ETB			-0.2274(-1.7329, 1.2782)	0.7029	0.1518 (-1.4445,1.7482)	0.8208
	>1500 ETB			0.4968(-0.8328, 1.8264)	0.2853	0.7272 (-0.6974, 2.1518)	0.1731
9	Muslim			0.0272(-1.2267, 1.2812)	0.9475	-0.1565 (-1.4740, 1.1611)	0.7318
	Protestant			-0.0811(-1.3294,1.1673)	0.8432	-0.4973 (-1.8713,0.8768)	0.3166
	Catholic			0.5287(-1.3728, 2.4301)	0.5782	0.2043 (-1.8961,2.3047)	0.8602
	Others			1.8690(-0.0686, 3.8066)	0.0584	0.7676 (-1.3592,2.8944)	0.5187
**Random Effects**							
Intercept standard deviation		0.6238		0.4478		2.0665	
Age- slope standard deviation						0.0982	
Gender-slope standard deviation						2.3424	
Preference of service fees with discount slope standard deviation						2.6259	
Preference of service fees with free of charge slope standard deviation						1.5087	
${\rho }_{01}$ Intercept-Age slopes correlation						-1.00	
${\rho }_{02}$ Intercept-Gender slopes correlation						0.25	
${\rho }_{03\left(1\right)}$ Intercept- Preference of service fees with discount slope correlation						0.78	
${\rho }_{03\left(2\right)}$ Intercept- Preference of service fees with free of charge slope correlation						0.44	
${\rho }_{12}$ Age-Gender slopes correlation						-0.25	
${\rho }_{13\left(1\right)}$ Age- Preference of service fees with discount slopes correlation						-0.78	
${\rho }_{13\left(2\right)}$ Age- Preference of service fees with free of charge slopes correlation						-0.44	
${\rho }_{23\left(1\right)}$ Gender- Preference of service fees with discount slopes correlation						-0.41	
${\rho }_{23\left(2\right)}$ Gender- Preference of service fees with free of charge slopes correlation						-0.76	
${\rho }_{33\left(12\right)}$ free of charge - with discount (Preference of service fees) slopes correlation						0.90	
AIC Score		483.8		378.7		370.8	

Model 1: null two-level model; Model 2: random intercepts two-level model; Model 3: random slopes two-level model; No 1–9 sequentially refers variables name that are listed in Table 3; the categories No, Formal employee, Male, < 2,500 ETB, Urban, At usual rate, < 250 ETB and Orthodox respectively are the corresponding reference category for variables name that are listed in Table 3 from No 2–9 ; CI: confidence interval

At 5% level of significance, the intercept, awareness, gender, preference of service fees (with free of charge) and student’s monthly average income (251–500 ETB) were significant predictor variables both in model 2 and 3. In model 2, in addition to the above covariates, parent’s occupation (causal laborer) and preference of service fees (with discount) were significant. Among all considered models in this study the AIC score for model 3 (370.8) is smallest. This means that relatively this model best fitted the dataset. So that future descriptions are based on this model.

The odds ratio for awareness of RHS is exp(3.2565) = 25.99. This means that, in a comparison of two students with different awareness of RHS but the same values on the remaining eight covariates and college/school average risk (i.e., the value of the random effect), the student with awareness of RHS has 2.12 times higher odds of utilizing RHS than the student with no awareness of RHS. Put another way, the odds of utilizing RHS for student who is aware of RHS is exp(3.2565) = 25.99 times higher than the odds of utilizing RHS for the student who is not aware of RHS when comparing two subjects within the same college/school who differ in their awareness of RHS but who share identical values of the remaining eight covariates.

Similarly, the odds of utilizing RHS for female student is exp(2.8215) = 16.8 times higher than the odds of utilizing RHS for male student when comparing two students within the same college/school who differ in their gender but who share identical values of the remaining eight covariates. Again, the odds ratio for free of charge (category of preference of service fees) is exp(-1.5117) = 0.22. This means that, for student whose preference of RHS fees is free of charge, the odds of utilizing RHS are 0.22 times lower than for student whose preference is at usual rate when comparing two students within the same college/school who differ in their preference of RHS fees but who share identical values of the remaining eight covariates. In other words, student whose preference of RHS fees is free of charge is 78% (1-0.22 = 0.78) less likely to utilize RHS compared to student whose preference is at usual rate controlling the values on the remaining eight covariates and college/school average risk. exp(2.2592) = 9.58 is also the odds ratio of 251–500 ETB (one of the categories of student’s average monthly income). This is also reveals that, in a comparison of two students with different average monthly income but the same values on the remaining eight covariates and the value of the random effect, the student with an average income of 251–500 ETB has 9.58 times higher odds of utilizing RHS than the student with an average income less than 251 ETB.

The correlation between slopes of; age and gender, age and preference of service fees (with its both categories), and gender and preference of service fees (with its both categories) is negative. This implies that the slopes of each above paired covariates tend to vary in opposite directions across colleges/schools. Stated differently, colleges/schools exhibiting a greater slope for one predictor variable (e.g., age) typically exhibit a lower slope for the other (e.g., gender). Specifically, it means that if the effect of age on utilization of RHS is positive in any particular college/school, then the effect of gender on utilization of RHS will be negative on this particular college/school. The correlation between the random intercept and random slopes of each covariate (i.e., age, gender and preference of service fees (with its both categories)) is positive. This implies that the slopes on each of these covariates tend to be steeper for students with higher intercepts. The standard deviation of the random slope on gender, preference of service fees (both with discount & free of charge) and random intercept are 2.3424, 2.6259, 1.5087 and 2.0665 respectively. These figures imply that slopes on these covariates and the intercepts show higher variability across different colleges or students, revealing the present of heterogeneity in the dataset. In other words, it means that; on average there is different level of RHS utilization across colleges/schools or on students due to more dispersed intercepts and the relationship between the each of these covariates and utilization of RHS differs across different colleges/schools or students due to presence of more dispersed slopes on these covariates (Table 4).

## Discussion

The findings from this study emphasize the crucial of using a two-level random slope model to model the determinant of RHS utilization among Assosa University regular class students. In line with previous study, this study revealed that the covariates; student’s awareness about RHS, gender, preference of service fees and student’s monthly average income were significant predictor variables of RHS utilization [8, 30–32]. In this study findings, students who; preferred service fee as usual rate, have awareness about RHS, are females and have high monthly average income were more likely to utilize RHS. This finding also consistent with findings of [22, 33, 34].

The implication of these findings can be expressed by two ways. First, further research in the future should be conducted by figuring out the precise mechanisms through which the study’s factors affect the use of RHS, recognizing the justifications for some students’ preference to pay RHS at a rate comparable to the standard rate and creating efficient interventions to boost RHS use in the students who stand to gain the most from them. Second, public health policy and practice to be improved by; providing intervention mechanism that was directed towards students who are unaware of RHS, male students, and students from lower-income families; carried out campaigns to raise awareness of students regarding RHS and offered RHS at reasonable costs.

Contradicted to the present study findings, female students were less likely to utilize RHS than male students in [10, 23, 35] study findings. The difference may be due to the presence of differed cultural settings of these study area from the present study. Moreover, these studies were carried out in a particular cultural setting where male students are more inclined to look for RHS.

The findings from this study also inconsistent from study findings [36, 37] in which they found that compared to students with higher income levels, those with lower income levels were more likely to use RHS. This difference may due to the fact that these studies were conducted in rural settings. Students with low average income from this area have an access of services with subsidies or a community-based initiative which in turn helps them to afford RHS cost. Indirectly, this findings support the findings of present study in the way that students with high monthly average income are more likely to have access to RHS and to be afford of the cost for these services. According to [30–32, 38] study findings, students who were aware of RHS were more likely than those who were not to make use of these services. This is also another consistent result from the present study finding. This is because, those students who have awareness about RHS may be more likely to look for and use them. In contrary, those who haven’t might not be aware that these services are available or might not know how to use them. In general, this highlights the significance of thorough education programs on reproductive health that support accurate information and favorable attitudes toward reproductive health services.

## Conclusion

In this study, students who; preferred service fee as usual rate, have awareness about RHS, are females and have high monthly average income were more likely to utilize RHS. RHS utilization among undergraduate regular students in Assosa University is likely to increase more effectively with interventions that address these factors.

### Electronic supplementary material

Below is the link to the electronic supplementary material.

Supplementary Material 1

## Data Availability

The data have been released upon request of the corresponding author.

## Abbreviations

RHS: Reproductive health service
STD: Sexual Transmitted Disease
HIV: human immunodeficiency virus
AIDS: Acquired Immune Deficiency Syndrome
SRS: Simple Random Sampling
SYS: Systematic Sampling
PPS: Population Proportion to Size
ETB: Ethiopian Birr
PPH Test: Population Proportion Heterogeneity Test
AIC: Akaike Information Criterion
