# Supplementary material for: Utilization of reproductive health services among undergraduate regular class students in Assosa University, BGRS: a comparison among three varieties of multilevel binary logistic regression models
Source: BMC Health Serv Res. 2024 May 26;24:664. doi: 10.1186/s12913-024-11123-8 (PMC11129371; doi:10.1186/s12913-024-11123-8)

**Assosa University**

**College of Natural and Computational science**

**Department of Statistics**

**Questionnaire**

**Dear Respondent:**

This questionnaire is designed to collect data about the socio-economic, demographic, health and socio-cultural factors of utilization of reproductive health services among Assosa University undergraduate regular class students. You have been selected to complete the questionnaire as part of a sample for the study. I would very much appreciate your participation in this study.

The data collected from this questionnaire will be used for the purpose of the research. Your genuine responses are very important in order to meet the purpose of the study. To this end, whatever information you will provide will be kept strictly confidential and will not be shown to other persons.

**Instruction: Tick your choice for each question**

1. How old are you?________ years.
2. Do you have awareness about the reproductive health services?

Yes No
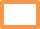


1. Do you utilize reproductive health services?

Yes
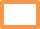
 No
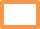


1. What is your Parents’ (father/mother) occupation?

Formal employee
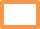
 Farmer
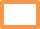
 Casual laborer
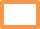
 Self-employee
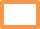


1. What is your sex? Male
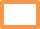
 Female
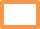

2. How many is your monthly average incomes?

<250 ETB
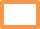
 251-500 ETB
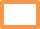
 501-1000 ETB
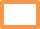


1001-1500 ETB
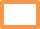
 >1500 ETB
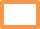


1. Where is your place of residence? Urban
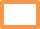
 Rural
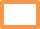

2. What is your Preference of service fees for reproductive health service?

At usual rate
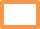
 With discount
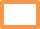
 Free of charge
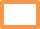


1. How many is your parents’ (father) monthly average incomes?

<2,500 ETB
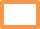
 2,501-4,000 ETB
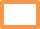
 4,001-5,000 ETB
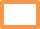


5,001-10,000 ETB
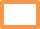
 >10,000 ETB
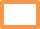


1. What is your religion?

Orthodox
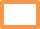
 Muslim
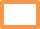
 Protestant
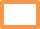
 Catholic
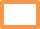
 Other
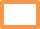

Supplement: Supplementary file 1 — Supplementary Material 1 [file 12913_2024_11123_MOESM1_ESM.docx]
